# Cerebellar dysfunction and autism spectrum disorders – what do we know?

**DOI:** 10.1192/j.eurpsy.2023.551

**Published:** 2023-07-19

**Authors:** C. Pinheiro Ramos, M. Alves, J. Marta, R. Ribeiro, A. Gamito

**Affiliations:** Psychiatry and Mental Health Department, Setúbal Hospital Center, Setúbal, Portugal

## Abstract

**Introduction:**

Autism spectrum disorders (ASD) are complex neurodevelopmental conditions characterized by impairments in social cognition and repetitive behaviors with onset in early infancy. Deficits in emotion recognition, social perception, and communication have been identified as core symptoms of ASD.

Comorbid disorders are frequent, namely psychiatric illness, epilepsy, sleep disruption, and hyperactivity.

Immune profile changes during early life may contribute to pathogenesis of ASD. Other risk factors include advanced parental age, fetal environment, fertility treatments, medications, and nutritional and toxic factors.

Several brain regions are involved in the pathophysiology of ASD but the cerebellum is the structure most consistently found altered. An increased risk of ASD is associated with cerebellar damage.

**Objectives:**

To highlight the importance of understanding the key processes of cerebellar development and how altered cerebellar function leads to social and cognitive impairments, and consequently ASD.

**Methods:**

Non-systematic review of the literature using *Pubmed* database. Papers were selected according to their relevance.

**Results:**

From imaging studies, we can understand that cerebellum is not just about motor function. Different tasks like adding working memory, emotional and social processing, and language seem to be part of core functions of the cerebellar circuit.

Adults with lesions in the cerebellum can develop cerebellar cognitive affective syndrome (CCAS), with core symptoms of impaired executive function, difficulties in spatial cognition, blunted affect, or inappropriate behavior. Some children who have tumor resection surgery for medulloblastomas also exhibit symptoms of CCAS, and some experience posterior fossa syndrome (PFS).

The linguistic, cognitive, and behavioral deficits in CCAS and PFS may contribute to explaining how cerebellar alterations are related to ASD, which is a neurodevelopmental disorder characterized by an earlier onset and broader spectrum of these symptoms.

**Conclusions:**

The literature has suggested an important role for cerebellar dysfunction in etiology of ASD, under certain premises: (a) cerebellar expansion temporarily coincides with onset of ASD; (b) cerebellum is prone to lesions during this period; (3) cerebellar lesions contribute to dysfunctional social and language abilities.

Disturbances in cerebellar development lead to alterations in higher cognitive functions, due to changes in Purkinje cells. These dysfunctional neurons, once integrated into a brain circuit that controls complex tasks, lead to these functions becoming aberrant.

It is therefore fair to say that cerebellum is important for development of the so-called “cognitive and social brain” since it is itself part of this network. So, the cerebellum certainly plays a relevant role in pathophysiology of ASD.

**Disclosure of Interest:**

None Declared

